# Commentary: Correlation analysis of metabolic parameters influencing postoperative tendon healing after Achilles tendon rupture repair

**DOI:** 10.3389/fmed.2026.1970521

**Published:** 2026-10-23

**Authors:** Filippo Spiezia, Francesco Oliva, Filippo Migliorini, Nicola Maffulli

**Affiliations:** 1 Department of Health Sciences, University of Basilicata, Potenza, Italy; 2 Department of Orthopaedic and Trauma Surgery, Ospedale San Carlo, Potenza, Italy; 3 Department of Orthopaedic and Trauma Surgery, Università Degli Studi di Roma San Raffaele Telematica, Rome, Italy; 4 Department of Trauma and Reconstructive Surgery, University Hospital of Halle, Martin-Luther University Halle-Wittenberg, Halle (Saale), Germany; 5 Department of Life Sciences, Health, and Health Professions, Link Campus University of Rome, Rome, Italy; 6 Department of Orthopaedic and Trauma Surgery, Eifelklinik St. Brigida, Simmerath, Germany; 7 Department of Trauma and Orthopaedic Surgery, Faculty of Medicine and Psychology, University La Sapienza, Rome, Italy; 8 Centre for Sports and Exercise Medicine, Barts and the London School of Medicine and Dentistry, Mile End Hospital, Queen Mary University of London, London, United Kingdom; 9 School of Pharmacy and Bioengineering, Keele University School of Medicine, Stoke-on-Trent, United Kingdom

**Keywords:** Achilles tendon rupture, hyperuricemia, magnetic resonance imaging, metabolic disorders, rehabilitation, signal-to-noise quotient, tendon healing, uric acid

Ye and colleagues report that preoperative serum uric acid predicts the signal-to-noise quotient (SNQ) measured on T2-weighted MRI three months after open repair of acute Achilles tendon rupture, and that hyperuricaemic patients show markedly higher SNQ values than normouricaemic controls (1). They pose an excellent clinically relevant question, and the application of a quantitative imaging endpoint to metabolic status after Achilles repair is a genuine addition. We would like to raise four points that, in our view, qualify the strength of the conclusion, as well as one point from the prior literature.

The first concerns rehabilitation. The protocol described involves a long-leg cast in 20–30 degrees of plantarflexion with strict non-weight-bearing for 4–6 weeks, an adjustable brace from 6 to 12 weeks, and resistance training only after 12 weeks. The authors themselves cite the randomised evidence that functional weight-bearing mobilisation enhances the early healing response after Achilles rupture (2). Our own comparative work on a slowed-down rehabilitation timeline, in which wedge removal, boot discontinuation and the introduction of resisted and eccentric loading were each deferred, produced significantly better tendon resting angle and strength symmetry at one year (3); however, that cohort underwent percutaneous repair in which both groups began weight-bearing early after surgery, so it is not fully comparable with the open-repair cohort studied here. Loading history is therefore not a neutral background variable but a determinant of tendon healing, and the three-month assessment falls precisely where cumulative loading histories diverge most. No data on weight-bearing progression or rehabilitation adherence are presented. Since patients with hyperuricaemia commonly carry additional metabolic comorbidity, differential progression through an immobilisation-heavy protocol represents a plausible source of residual confounding for the observed SNQ difference, rather than a demonstrated alternative explanation. We would encourage the authors to report any available loading or adherence data, since these would materially strengthen the causal argument.

The second concerns the endpoint itself. SNQ has been validated principally in the assessment of ligament graft maturation, and its application to a repaired Achilles tendon rests on the assumption that higher T2 signal at three months indicates a less mature repair rather than a normal stage of the reparative process. That assumption is not fully established for tendons. Elevated signal at this stage may equally reflect an active, well-vascularised reparative response rather than failed healing, and the direction of the association cannot be resolved without considering clinical outcome. Achilles-specific data are, in fact, beginning to accrue on this point: in one longitudinal MRI study of repaired Achilles tendons, patients with poor tendon-stump coaptation had significantly higher SNQ at three months than those with good coaptation, a difference that had resolved by six and twelve months despite similar final AOFAS scores, ATRS and muscle strength between groups; the authors interpreted the early elevation as a relative delay in tissue maturation rather than a failure to heal (4). This distinction matters for the present study: SNQ characterises the maturation status of the local repair tissue, whereas patients’ symptoms, activity capacity and the macroscopic function of the muscle-tendon unit, which are also shaped by pain, muscle atrophy and rehabilitation progress, are better captured by ATRS, AOFAS, the tendon resting angle and strength testing. The study reports no functional scores, no tendon resting angle, and no re-rupture data, so until SNQ is shown to track outcomes that matter to patients after Achilles repair — as distinct from the appearance of the local repair tissue alone — it remains an uncertain surrogate for a patient's overall healing status.

The third concerns an inconsistency between the discussion and the conclusion. In the discussion, the authors appropriately state that uric acid has limited predictive value, that it should serve only as an adjunctive risk predictor, and that treatment decisions should not rest on uric acid levels alone. The abstract and conclusion nonetheless recommend perioperative uric acid control in hyperuricaemic patients. An association observed in 60 patients in a single-centre retrospective observational study cannot support a treatment recommendation, and the more measured language of the discussion should prevail. The randomised trial of urate-lowering intervention that the authors propose is the correct next step, and the recommendation should await it.

The fourth concerns the analysis. The multivariable model carries nine parameters in 60 patients and explains little variance, with an adjusted R^2^ of 0.114. Lipid values were missing for a quarter of the cohort and were imputed. Six markers were tested by rank correlation without adjustment for multiplicity, and the one marginal result, namely total bilirubin at *p* = 0.042, duly disappears in the regression. Fifty-eight of the 60 patients were male, and the hyperuricaemic group was male in its entirety. The findings derive predominantly from male patients, and their generalisability to female patients is very limited. The uric acid signal survives all of this and is more credible, but the model should be presented as hypothesis-generating rather than predictive.

Finally, the authors state that the relationship between systemic metabolic function and postoperative tendon healing has been little explored. We would respectfully point out that, in a multicentre study of 340 patients treated surgically for Achilles tendon rupture across five Italian centres, the presence of a metabolic disorder independently and negatively predicted return to work and sporting activity (5). That work used functional rather than imaging endpoints, which is precisely why the present contribution is worthwhile: it supplies a structural correlate for an association already documented clinically. Framed in that way, the finding gains rather than loses standing.

None of this detracts from a carefully conducted study addressing an understudied issue in this field. The mechanistic case that urate disrupts tendon stem and progenitor cell function is coherent (6), and the clinical conundrum reported here is worth pursuing. A prospective design incorporating T2 mapping, documented loading progression, functional outcomes and a balanced sex distribution would settle what the present data can only suggest.
